# Who uses pharmacy for flu vaccinations? Population profiling through a UK pharmacy chain

**DOI:** 10.1007/s11096-016-0255-z

**Published:** 2016-01-28

**Authors:** Claire Anderson, Tracey Thornley

**Affiliations:** School of Pharmacy, University of Nottingham, Nottingham, NG72RD UK; Boots UK, Nottingham, UK

**Keywords:** Access, Community pharmacy, Deprivation, Immunization, Influenza, Risk groups, United Kingdom, Vaccination

## Abstract

*Background* There is a need to increase influenza vaccination rates in England particularly among those who are under 65 years of age and at-risk because of other conditions and treatments. *Objective* To understand the profile of people accessing flu vaccination services within a large pharmacy chain. *Method* Pharmacists requested people who had been vaccinated in 2014/15 to complete a questionnaire. Data was captured electronically on vaccine delivery levels across 1201 pharmacies. Deprivation profiles were calculated using the Carstairs index. *Results* 1741 patients from a total of 55 pharmacies completed the survey. Convenience and accessibility remain the key reasons for attending pharmacy. Pharmacy services are accessed by people from all postcode areas, including some from the most deprived localities. *Conclusion* Pharmacy flu vaccination services complement those provided by general practitioners to help improve overall coverage and vaccination rates for patients in at-risk groups. These services are highly accessed by patients from all socio demographic areas, and seem to be particularly attractive to carers, frontline healthcare workers, and those of working age.

## Impact of findings on practice

- Pharmacy flu vaccination services are highly accessed by patients from all socio demographic areas, and seem to be particularly attractive to carers, frontline healthcare workers, and those of working age.
- The timings show that access for flu vaccination is likely to be due to the convenience and accessibility of pharmacies.

## Introduction

Influenza (flu) vaccination can prevent death and ill health and reduce hospital admissions. There is a need to increase flu vaccination rates in England particularly among those who are <65 years of age and at-risk because of other conditions and treatments. Patients in the following at-risk groups are eligible for free vaccination on the National Health Service (NHS) in England: aged 2–4 years or ≥65 years; pregnant women; long stay care home residents; in receipt of a carer’s allowance; chronic disease patients; and health care professionals. Despite this, not everyone who is eligible gets vaccinated.

NHS flu vaccinations are available in primary care through doctor’s surgeries and have recently been nationally commissioned for the first time in community pharmacies (2015/16 flu season). Prior to this, vaccination services were commissioned locally by NHS teams. People who are not eligible for free flu vaccinations on the NHS are able to pay privately to access the service through pharmacies During the 2013/14 ‘flu vaccination’ campaign in England only around 52 % of at-risk patients were vaccinated against a target of 75 %. In the 2014/15 season, just 50.3 % patients in the at-risk group had been vaccinated by the end of January 2015 and only 44.1 % of pregnant females (www.gov.uk/government/uploads/system/uploads/attachment_data/file/319682/2902502_FluVaccineUptake_HCWs_2013-14_acc.pdf). In the areas where flu vaccinations were commissioned through community pharmacies there was a higher uptake among <65 s and at-risk groups than in the four areas that did not commission the service (www.england.nhs.uk/wp-content/uploads/2015/04/05-amr-lon-antimicrobial-stewardship.pdf).

Providing vaccinations through additional providers increases the overall vaccination rates especially in harder to reach groups, such as patients who are eligible but who have not been previously vaccinated and those at-risk [1]. A study in West Yorkshire found that community pharmacies also extended the reach of vaccination; 16.8 % of the 8046 people accessing the service had not had a flu vaccination previously [2].

People with long-term conditions who are in at-risk groups will normally visit a pharmacy up to five times during the flu season, presenting opportunities for pharmacists to encourage vaccination. Over a quarter of a million private flu vaccinations have been successfully provided by community pharmacies in England and Wales in one scheme alone [3]. Tower Hamlets Clinical Commissioning Group (CCG) exceeded the national target for 2013/14 in those ≥65 years by achieving 76 % vaccination. In a pan-London scheme, 11 % of all vaccinations in this age group were undertaken by community pharmacies [4]. Data collected for one NHS locality in Scotland indicated that over 13 % of their at-risk cohort vaccinated by pharmacies were pregnant [5].

Evaluation of existing flu vaccination services provided by community pharmacists shows that patients strongly welcome the additional choice [1–6]. While people at-risk can receive flu vaccinations free via the NHS, some choose to pay privately because they perceive that community pharmacy access is easier [6]. In a study of 3500 patients, 99 % of patients rated the flu vaccination service as above average or excellent and 20 % said they would not otherwise have been vaccinated [7].

UK studies indicate that accessibility and convenient opening times encourage the use of pharmacies [1–6]. Findings are similar in other countries; in a Canadian trial participation in community pharmacy schemes was found to be due to convenience. In a recent pharmacy survey of 1502 people receiving flu vaccines in four community pharmacies in Canada, convenience and accessibility were major factors for accessing the vaccination [8]. A quarter of those vaccinated were not regular vaccination recipients, 28 % would not have had flu vaccination otherwise and 21 % of these were high-risk patients.

Pharmacies open for longer hours than general practice (GP) surgeries and most are open at weekends. In West Yorkshire where pharmacies delivered 8046 flu vaccinations, the peak times for vaccination were mid-morning and mid-afternoon, with 7.7 % vaccinations being delivered on a Saturday or Sunday and 2.5 % consultations being delivered out of hours on a weekday (before 8 am or after 6 pm) [2]. This trend was mirrored in an American study which also found that in particular, younger, working-aged, healthy adults accessed a variety of vaccinations during off-clinic hours [9].

A recent US study indicated that those who received a flu vaccine in non-medical settings were more likely to have attained a higher education, not have high-risk conditions, have a primary care doctor, or have had a routine check-up in the previous 12 months [10]. However, little is known about the demographics of people who attend pharmacy for flu vaccinations in England.

## Aim of the study

The aim of our study was to understand the profile of people accessing flu vaccination services within a large community pharmacy chain, and whether vaccination services provided by community pharmacies are addressing the health inequalities agenda. The secondary objective was to provide data to support local and national discussions for commissioning of NHS flu vaccination services in community pharmacy.

## Ethical approval

As this was a service evaluation, and all data was anonymised ethical approval was not required.

## Methods

This paper discusses retrospective data collected through a sample of pharmacies in England during the 2014/15 flu season. Pharmacies were part of the largest chain of community pharmacies in the UK and were well distributed across the country. The chain encompasses those which serve small local communities (including some of the most deprived locations in the country), health centres, high streets and large retail shopping centres.

Pharmacists were requested to ask every person who had a vaccination between October 2014 and March 2015 to complete a questionnaire. A sample of data was captured electronically on the delivery levels of NHS and private flu vaccinations.

Data was analysed in Microsoft Excel 2007, and deprivation profiles calculated using the Carstairs index (used to calculate deprivation quintiles for least and most deprived); based on four census indicators: low social class, lack of car ownership, overcrowding, and male unemployment. A negative value indicates areas of low deprivation, and a positive value equates to high deprivation. Respondents were also asked to state their highest level of education.

Survey responses were categorised into those who were NHS eligible, and those who were not (classified as private patients). NHS eligible patients were further sub-divided into those who accessed the NHS service, and those who chose to pay privately (despite being NHS eligible). There were also a number of patients who were classified as NHS eligible, but it was not clear whether they accessed the private or NHS service (classified as unknowns). Where fields are missing on the forms, this was classed as ‘missing data’.

## Results

Data were recorded electronically by pharmacists for 150,997 vaccinations across 1201 pharmacies in England. A slightly higher proportion of NHS vaccinations were recorded during the week (83.6 %) compared to privately funded vaccinations (79.6 %). The majority (85.6 %) of all flu vaccinations were recorded between 9am and 5 pm, peaking slightly between 11am and 1 pm (24.0 %). A slightly higher proportion of vaccinations were carried out before pre-9 am) and after 7 pm during the week (4.8 %) compared to the weekend (2.3 %). Data source: electronic data (n = 150,997). This is likely to reflect the pharmacy opening times as well as the behaviour of patients accessing these services.

Complete surveys were collected from 1741 patients from 55 pharmacies conducting private and NHS vaccinations across three localities representing 18.9 % of vaccinations that were carried out within these pharmacies.

Patients are eligible for a free NHS vaccination if they are aged ≥65 years, or in an at-risk category. Of all the forms returned, 30.8 % were for patients who were eligible for an NHS vaccine and of these, 25.6 % paid privately (10.8 % unknown, 63.6 % accessed the NHS service). Data source; surveys including complete eligibility details (n = 1683).

Data on gender were available for 1736 patients and indicated that 64.6 % of the sample were female. This differed slightly for patients who were NHS eligible but who chose to pay privately, where the percentage of females rose to 71.5 %.

The median age of patients accessing all services was 50 (inter quartile range 22). Half (49.7 %) of patients accessing the NHS service were >65. The profile of patients accessing the private service was younger: 20.6 % were aged between 35 and 44, compared to 14.8 % NHS eligible paying privately, and 11.2 % NHS. Over half of patients accessing the private service were aged between 45 and 64 (58.2 %). Of those that were NHS eligible but paid privately 50.4 % were also within this age range, of which 61.8 % fell in the 55–64 year-old category. Data sourced from surveys which included both eligibility and age fields (n = 1668).

Patients’ types of NHS eligibility are listed in Table 1. The percentage of patients with long-term conditions accessing flu vaccinations was similar across the NHS service (36.9 %), and those who were NHS eligible but paid privately (38.9 %). Of those that were NHS eligible and paid privately, 36.7 % were carers or healthcare/social-workers.

**Table 1:** NHS eligibility (data sourced from survey, n = 453)

Risk group	All NHS eligible paid privately N = 453	%	NHS eligible paid privately N = 90	%	NHS service patients N = 312	%	Unknown no of patients N = 51	%
Aged 65+	207	45.7	21	23.3	168	53.8	18	35.3
Pregnant	10	2.2	1	1.1	9	2.9	0	0.0
Long term condition	166	36.6	35	38.9	115	36.9	16	31.4
Carer	27	6.0	16	17.8	5	1.6	6	11.8
Healthcare worker/social care worker	43	9.5	17	18.9	15	4.8	11	21.6
Missing data	83		47		29		7	
Total	536	100.0	137	100.0	341	100.0	58	100.0

A higher education or professional qualification was attained by 48.8 % of patients who were NHS eligible and paid privately, compared to 37.5 % of those who accessed the NHS service. The lowest proportion of patients to have no qualifications were those that paid privately (5.2 %), compared to 16.5 % of NHS patients and 15.7 % of NHS eligible patients that paid privately (data source: surveys with both education and eligibility fields [n = 1617]).

**Fig. 1:**
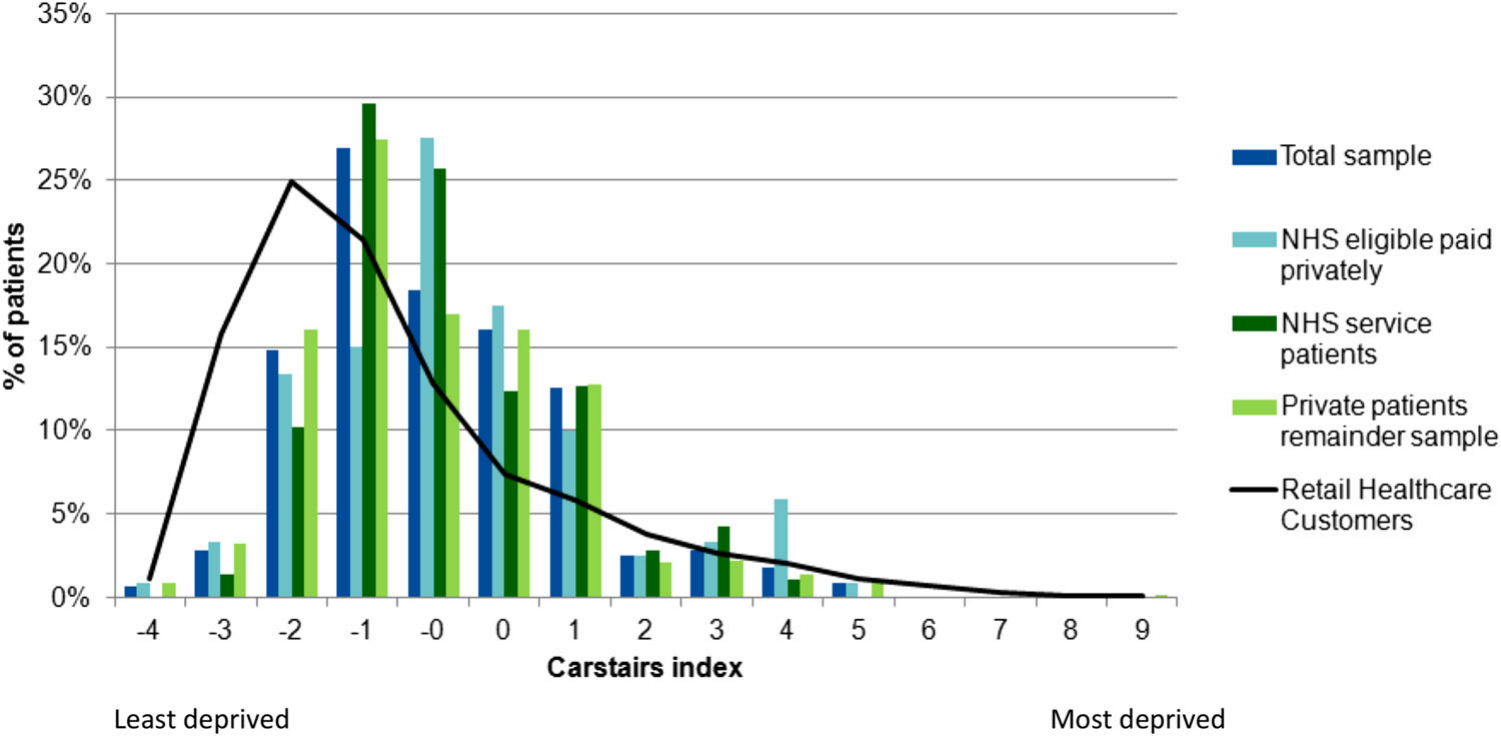
Carstairs index for patients accessing NHS and private flu vaccinations (survey data n = 1432)

Patients from all demographic areas accessed both the NHS and private service within the pharmacy. Comparing this to the Carstairs profile of patients who normally visit a pharmacy ( Fig. 1) shows that the service was accessed more by patients from deprived areas.

Every patient cited convenience or accessibility as the primary reason for why they chose to receive their flu vaccination from a pharmacy, regardless of whether it was NHS or privately funded. For those patients who were NHS eligible but paid privately, 16.7 % accessed through pharmacy because of preference, 8.3 % through use of an employer-supported scheme and 6.1 % through personal recommendation. Data source: surveys which included details of both eligibility and reason for accessing the service (patients could choose multiple reasons, n = 1643).

## Discussion

This paper builds on our previous publication [6] and clearly shows that community pharmacies can work alongside GP surgeries and thus vaccinate more at-risk patients. As with previous studies convenience and accessibility remain the key reasons for attending pharmacy for the flu vaccination [4–8]. This is true for people of all ages, not just those likely to be in employment. Even some patients eligible for the free vaccination chose to attend pharmacy and pay. This could be due to restrictions around service delivery within particular localities, limited eligibility criteria or operating times. Although more women accessed the service, men, who can be particularly difficult to reach, did receive their flu vaccinations in pharmacies.

Of those who were NHS eligible but paid privately, 36.7 % were health professionals or carers. A UK study found that significantly more carers were vaccinated through pharmacies than GP [4]. Community pharmacy also appears to be catering for those who find it less easy to access a GP surgery.

Carstairs data indicates that pharmacy based flu vaccination services are accessed by people from all postcode areas, including some from the most deprived areas, thus helping to address the health inequalities agenda. Typically those with higher education are the largest users of pharmacy flu vaccination services [10]; however, this analysis demonstrates that the service was accessed by patients from a broad spectrum of educational backgrounds.

## Conclusions

Pharmacy flu vaccination services complement those provided by GPs to help improve overall coverage and vaccination rates for patients in at-risk groups. These services are highly accessed by patients from all socio-demographic areas, and seem to be particularly attractive to carers, frontline healthcare workers, and those of working age. The timings of access show that this is likely to be due to the convenience and accessibility of pharmacies. There is an opportunity for pharmacists to support vaccination services further by targeting those that are unlikely to attend GP surgeries, as well as those with long-term conditions on repeat medicines who are eligible for a NHS vaccine.
